# Exploring Endothelial Colony-Forming Cells to Better Understand the Pathophysiology of Disease: An Updated Review

**DOI:** 10.1155/2022/4460041

**Published:** 2022-05-16

**Authors:** Qiuwang Zhang, Anthony Cannavicci, Michael J. B. Kutryk

**Affiliations:** ^1^Division of Cardiology, Keenan Research Center for Biomedical Science, St. Michael's Hospital, Unity Health Toronto, University of Toronto, Toronto, ON, Canada M5B 1T8; ^2^Institute of Medical Science, Cardiovascular Sciences Collaborative Specialization, University of Toronto, Toronto, ON, Canada M5S 1A8

## Abstract

Endothelial cell (EC) dysfunction has been implicated in a variety of pathological conditions. The collection of ECs from patients is typically conducted postmortem or through invasive procedures, such as surgery and interventional procedures, hampering efforts to clarify the role of ECs in disease onset and progression. In contrast, endothelial colony-forming cells (ECFCs), also termed late endothelial progenitor cells, late outgrowth endothelial cells, blood outgrowth endothelial cells, or endothelial outgrowth cells, are obtained in a minimally invasive manner, namely, by the culture of human peripheral blood mononuclear cells in endothelial growth medium. ECFCs resemble mature ECs phenotypically, genetically, and functionally, making them excellent surrogates for ECs. Numerous studies have been performed that examined ECFC function in conditions such as coronary artery disease, diabetes mellitus, hereditary hemorrhagic telangiectasia, congenital bicuspid aortic valve disease, pulmonary arterial hypertension, venous thromboembolic disease, and von Willebrand disease. Here, we provide an updated review of studies using ECFCs that were performed to better understand the pathophysiology of disease. We also discuss the potential of ECFCs as disease biomarkers and the standardized methods to culture, quantify, and evaluate ECFCs and suggest the future direction of research in this field.

## 1. Introduction

Endothelial progenitor cells (EPCs) were first isolated from human peripheral blood as a subset of CD34^+^ mononuclear cells (MNCs) that were able to differentiate into endothelial cells (ECs) *in vitro* [1, 2] and incorporate into newly formed vessels *in vivo* [1]. Since their discovery over two decades ago, human EPCs have been extensively investigated as disease biomarkers or therapeutic agents by employing either circulating EPCs that are defined mostly as CD34^+^, VEGFR2^+^, and CD133^+^ MNCs in the blood or EPCs obtained through the culture of peripheral blood MNCs (PBMNCs) in endothelial growth medium [3–11].

Culture of human PBMNCs gives rise to two distinct types of EPCs, namely, early and late EPCs [12–20]. Early EPCs are obtained after ~7 days of culture [12–15]. They possess little proliferative capacity, secrete a variety of proangiogenic factors, and lack the ability to form tube-like structures [16–19]. In contrast, late EPCs emerge after two weeks of culture and are highly proliferative and have the capacity to form tube-like structures [18–24]. Late EPCs have also been termed as late outgrowth endothelial cells (OECs) [25, 26], endothelial outgrowth cells (EOCs) [27, 28], blood outgrowth endothelial cells (BOECs) [29, 30], and endothelial colony-forming cells (ECFCs) [31, 32]. Although CD3^+^CD31^+^ T lymphocytes and CD163^+^CD206^+^ macrophages have been found as part of early EPCs [33, 34], the majority of early EPCs are CD14^+^CD45^+^ [35]. In contrast, late EPCs are shown to be CD45^−^CD34^+^ [18, 19, 35]. Both early and late EPCs contribute to angiogenesis, but they act through distinct mechanisms. Early EPCs promote angiogenesis in a paracrine manner through the secretion of various growth factors [16–19], while late EPCs directly participate in the formation of new vasculature [20–23]. Transcriptional profiling demonstrated that early EPCs displayed a gene expression pattern of hematopoietic cells, suggesting that they are not endothelial progenitors, but rather hematopoietic cells with angiogenic capability, and that late EPCs expressed EC and progenitor cell markers and were considered to be “true” EPCs [18, 19]. With the emergence of these new findings, it became evident that the early and late EPC terminology is flawed. Therefore, a consensus nomenclature was proposed to use “myeloid angiogenic cells (MACs)” and “ECFCs” to replace early and late EPCs, respectively, that more precisely reflect the phenotypic and genetic characteristics of these cells [35].

The endothelium, composed of a monolayer of ECs, plays an essential role in the maintenance of vascular integrity and homeostasis through a variety of vasoactive substances, such as nitric oxide (NO), produced from L-arginine by endothelial NO synthase (eNOS) [36]. NO is predominantly responsible for the modulation of vascular tone through the induction of vasodilation. Other activities of NO include regulation of angiogenesis, inhibition of inflammation, and suppression of platelet adhesion and aggregation [36–39]. Endothelial dysfunction has been implicated in a number of pathological conditions, including atherosclerosis, hypertension, diabetes, venous thrombosis, and inflammatory diseases [40–43]. Currently, ECs from patients are collected postmortem or through invasive approaches such as surgery and interventional procedures [44–46]. ECs procured in these ways often reflect end-stage disease, limiting their value in deciphering disease evolution. ECFCs resemble ECs phenotypically, genetically, and functionally and can be procured in a minimally invasive manner. Additionally, genetic and epigenetic changes associated with diseases in ECs often exist in ECFCs, making ECFCs excellent surrogates for ECs.

## 2. Study of ECFCs to Help Decipher Disease Pathogenesis

Here, we provide a review of studies performed to better understand the pathophysiology of diseases using ECFCs (late EPCs, OECs, EOCs, and BOECs) isolated from the peripheral blood of patients.

### 2.1. Cardiovascular Diseases

#### 2.1.1. Coronary Artery Disease (CAD)

Hyperlipidemia has long been considered to be a main contributor to the development and progression of atherosclerosis [47–50]. By reviewing genetic, epidemiologic, and clinical evidence, the European Atherosclerosis Society Consensus Panel stated in 2020 that low-density lipoproteins (LDLs) cause atherosclerotic cardiovascular disease [50]. As a result of increased endothelial permeability caused by proatherogenic stimuli and cardiovascular risk factors, LDL cholesterol (LDL-C) and small dense LDL-C particles in particular migrate into the subendothelial space, where they accumulate and undergo modification to generate oxidized LDL, resulting in EC activation and subsequent infiltration of inflammatory cells, immune cells, and smooth muscle cells. The result is a cascade of events leading to the formation of the atherosclerotic plaque composed mainly of lipids, inflammatory cells, vascular smooth muscle cells, and extracellular matrix.

Although our understanding of the pathogenesis of atherosclerosis has significantly improved in the past decades, knowledge of how the endothelial barrier is impaired, how LDL passes through the endothelium, and how LDL is modified is limited [51, 52]. Discovery of ECFCs, as surrogates for ECs, opened new avenues for arthrosclerosis research. Besnier et al. hypothesized that ECFCs from patients with suspected CAD would reflect the endothelial function of the host, retain the phenotypic imprint of the disease state, and be used to uncover the mechanisms of atherosclerosis [53]. They found that ECFCs spontaneously grew in 178 out of a total of 828 patients with suspected CAD (21.5%). Multivariate analysis adjusting for age, sex, and clinical variables showed that hypertension was associated with a higher rate of successful ECFC growth, while obesity and obstructive CAD (diameter stenosis > 50% on CT angiography) had a lower rate of ECFC growth [53]. Functional assays revealed that ECFCs from those with confirmed CAD showed increased tube-like structure formation on Matrigel and elevated proliferation and migration rates compared with ECFCs obtained from those patients without CAD. Unexpectedly, the baseline levels of activated eNOS, Akt, or ERK1/2 that govern ECFC function and NO concentration were not found to be significantly different between CAD ECFCs and non-CAD ECFCs [53]. In view of the recognized role of oxidative stress in the pathogenesis of atherosclerosis, the authors measured the mitochondrial production of superoxide and showed that superoxide levels were positively correlated with the severity of CAD. It was concluded that patient-derived ECFCs likely maintained the *in vivo* disease state and could be applied to elucidate the mechanisms of atherosclerosis [53]. Increased ECFC proliferation in CAD patients was also described by Güven et al. and Tagawa et al. [5, 24]. Conversely, reduced CAD ECFC proliferation, migration, and tube-like structure formation were also described [54, 55], while Stroncek et al. observed similar proliferation rates, comparable tube-like structure formation capability, and NO production between CAD ECFCs and control ECFCs in 13 patients and 13 healthy controls [56].

In the search for the mechanistic link between miRNA dysregulation and ECFC dysfunction in CAD, Wang et al. employed RNA sequencing and identified over 200 dysregulated miRNAs in CAD ECFCs [55]. Of interest, a group of miRNAs targeting VEGF-A were found to be upregulated in CAD ECFCs, including miR-361-5p, miR-342-3p, miR-125b-5p, miR-34a-5p, miR-484, miR-103a-3p, miR-140-5p, and miR-125a-5p. The alteration of these 8 miRNAs in CAD ECFCs was further validated by RT-qPCR. Measurement of plasma miRNAs by RT-qPCR also revealed the elevation of miR-361-5p and miR-484, but not the other 6 miRNAs. The authors then analyzed activities of miR-484 and miR-361-5p in healthy ECFCs. Overexpression of miR-361-5p, but not miR-484, downregulated VEGF-A and impaired ECFC function. Of note, inhibition of miR-361-5p in CAD ECFCs using an antagomir restored cell function [55]. These results demonstrated the potential involvement of miRNAs in CAD pathogenesis.

The studies summarized above provide conflicting results, which may be mainly due to (1) different sample sizes ranging from 13 to 828 patients and (2) heterogeneity of patient populations with different degrees of CAD. Future studies that include a large patient population with stratification of disease severity are necessary.

#### 2.1.2. Hereditary Hemorrhagic Telangiectasia (HHT)

HHT, also termed Rendu-Osler-Weber Syndrome, is a genetic disorder characterized by vascular dysplasia, i.e., mucosal/dermal telangiectasia, and visceral arteriovenous malformations (AVMs), which can result in recurrent epistaxis, gastrointestinal bleeding, and stroke [57]. Mutations in the endoglin (*ENG*) and the activin receptor-like kinase 1 (*ACVRL1* also known as *ALK1*) gene cause type 1 and type 2 HHT (HHT1 and HHT2), respectively [58, 59], which are responsible for approximately 90% of all HHT cases [60]. Both genes encode receptors of the transforming growth factor-beta (TGF-*β*)/bone morphogenetic protein (BMP) signaling pathway that regulates EC function and vascular development [61, 62]. HHT is underdiagnosed and lacks an effective pharmacological therapy [60]. Currently, the mechanisms of HHT pathogenesis remain largely unknown.

**Figure 1:**
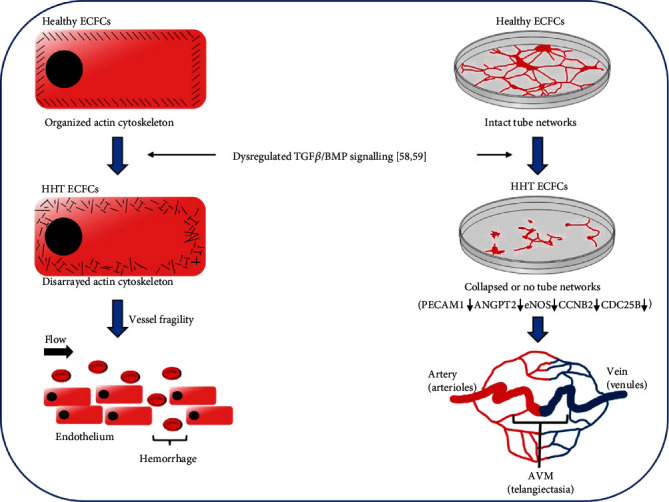
HHT ECFC abnormalities are compatible with clinical manifestations, e.g., epistaxis and vascular lesions, i.e., telangiectasia and AVMs seen in HHT patients.

ECFCs have been employed for research to better understand HHT pathophysiology [63, 64]. ECFCs from both HHT1 and HHT2 patients display disorganized actin cytoskeletons with extensive depolymerization of actin fibers (Figure 1), in contrast to the well-arrayed actin cytoskeletons with actin fibers crossing the entire cell seen in ECFCs from healthy subjects [63]. A disordered EC actin structure would predispose cells to rupture in response to changes in shear stress and blood pressure, leading to hemorrhage and the disappearance of capillary networks, both of which are characteristic of HHT (Figure 1). Endoglin, which is essential for the maintenance of an organized actin cytoskeleton in ECs [65], was found to be downregulated in HHT1 and HHT2 ECFCs [63]. Apart from the disordered cytoskeleton, HHT ECFCs also exhibited impaired ability to form tube-like structures [63].

It has been speculated that common target genes affected by abnormal TGF-*β*/BMP signaling in HHT1 and HHT2 play a role in HHT pathogenesis [64, 66]. Gene expression profiling has revealed that, compared with ECFCs from healthy donors, both HHT1 and HHT2 ECFCs expressed significantly lower levels of *eNOS*, angiopoietin-2 (*ANGPT-2*), platelet endothelial cell adhesion molecule-1 (*PECAM-1*), cyclin B2 (*CCNB2*), and cell division cycle 25B (*CDC25B*) [64]. These genes are well-known key players in vascular genesis and the maintenance of vascular homeostasis. NO is not only a potent vasodilator but also an EC survival factor [36]. Downregulation of eNOS reduces NO production, resulting in not only capillary EC apoptosis but also rupture due to the elevation of blood pressure [36]. PECAM-1 modulates EC migration and promotes the organization of ECs into vascular tubes [67, 68]. ANGPT-2, in combination with VEGF, stimulates the proliferation and migration of ECs and induces angiogenic sprouting of ECs [69, 70]. Cyclin B2 and CDC25B control cell cycle transition [71, 72] and their downregulation impede cell division, a process required for new vessel formation and stabilization. These genetic alterations correspond well to the vascular lesion phenotypes seen in HHT (Figure 1).

#### 2.1.3. Congenital Bicuspid Aortic Valve (BAV)

**Figure 2:**
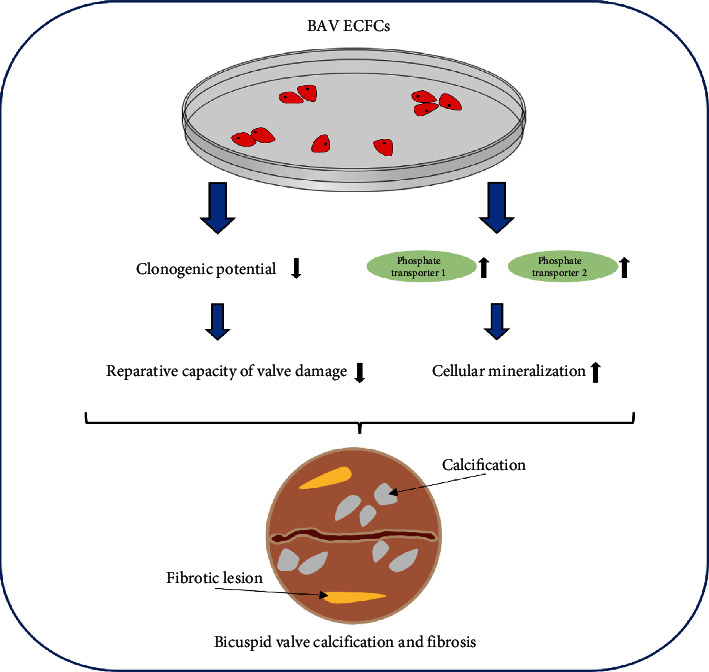
Reduced clonogenic potential and increased production of mineralization promoters (phosphate transporters 1 and 2) in BAV ECFCs may contribute to BAV degeneration.

BAV is the most common congenital cardiac abnormality, characterized by the presence of a two leaflet (bicuspid) instead of a tricuspid (three leaflet) aortic valve [73, 74]. Genetic, hemodynamic, and epigenetic factors are all thought to contribute to the progression of the valvulopathy (aortic valve stenosis and regurgitation) and aortopathy (aortic dilation and dissection) seen in BAV disease [73, 74]. There is recent evidence of endothelial cell dysfunction associated with BAV disease, although its relation to disease pathophysiology is poorly understood [75]. ECs from patients with BAV are usually surgically isolated intraoperatively during aortic valve/ascending aorta replacement surgery and have limited proliferative capacity, hampering efforts to clarify the role of endothelial dysfunction in disease onset and progression. Therefore, van de Pol et al. examined the function of ECFCs from BAV patients to elucidate EC involvement in BAV disease [76]. They successfully obtained ECFCs in 30% (3/10) of healthy individuals but only in 14.7% (5/34) of BAV patients and failed to isolate ECFCs in all patients with a dilated aorta. Although clonogenic potential was reduced (Figure 2), there were no significant differences in cell proliferation, cell size, or TGF-*β*-induced endothelial-to-mesenchymal transition between healthy and BAV ECFCs; under normal culture conditions, BAV ECFCs showed upregulation of phosphate transporters 1 and 2 (Figure 2), two potent osteogenic inducers that promote cellular mineralization [76]. These data suggest that BAV ECFCs, or possibly BAV ECs, are impacted and produce higher levels of osteogenic molecules that may contribute to BAV disease progression (Figure 2).

#### 2.1.4. Pulmonary Arterial Hypertension (PAH)

Pulmonary arterial hypertension (PAH) is a rare and devastating disease characterized by concentric intimal fibrosis and plexiform lesions in small pulmonary arteries, leading to arterial obstruction, elevation of pulmonary arterial resistance, increased pulmonary pressure, and eventually right heart failure. There are four major types of PAH: (1) idiopathic PAH (IPAH), formerly termed primary pulmonary hypertension with unknown etiology; (2) heritable PAH (HPAH), formerly known as familial PAH, which is caused in most cases by mutations in the BMP receptor type 2 gene (*BMPR2*) encoding BMPR-II; (3) drug- and toxin-induced PAH; and (4) PAH associated with other conditions such as congenital heart disease, connective tissue disease, and portal hypertension [77]. To date, the most studied types of PAH are IPAH and HPAH. Immunohistochemical analyses of lung samples collected postmortem or at lung transplantation from patients with IPAH have shown strong endothelial immunoreactivity and perivascular inflammatory cell infiltration in the plexogenic arteries [78]. Pulmonary arterial ECs (PAECs) isolated from explanted IPAH lungs have been shown to proliferate at a significantly higher rate than ECs from non-PAH controls [45, 79]. Reduced NO production in IPAH PAECs was also described [80]. Masri et al. observed persistent activation of signal transducer and activator of transcription 3 (STAT3) in IPAH PAECs [45]. Inhibition of STAT3 activation abrogated IPAH PAEC proliferation, suggesting that the STAT3 pathway is involved in the proliferative vascular lesions seen in IPAH lungs [45]. Taken together, endothelial hyperproliferation may have a critical role in IPAH pathogenesis.

Considering that endogenous EPCs can migrate into sites of vascular injury to participate in angiogenesis, Toshner et al. hypothesized that EPCs were involved in PAH pathogenesis [81]. To test this, they immunohistochemically analyzed explanted lung samples from patients with IPAH or HPAH and showed abundant CD133^+^ EPCs in plexiform lesions, which was in stark contrast to minimal CD133 signals detected in the control lung samples. Enumeration of circulating EPCs by flow cytometric analysis demonstrated that IPAH and HPAH patients had a significantly higher number of circulating EPCs (CD34, CD133, and VEGFR2 triple positive) than healthy controls [81]. These results led the authors to speculate that an increased number of circulating EPCs could migrate to, and engraft in, pulmonary arteries and further differentiate into ECs, to participate in the formation of plexiform lesions in patients with PAH [81]. Furthermore, HPAH ECFCs were isolated and characterized, and *in vitro* cell culture revealed that HPAH ECFCs had a significantly higher proliferative rate than control ECFCs [81]. More recently, Theilmann et al. examined the response to BMP9, an endothelial-selective BMPR-II ligand, of ECFCs from HPAH patients with confirmed *BMPR2* mutations. They reported that BMPR-II was reduced in HPAH ECFCs and BMP9 enhanced the proliferation of HPAH ECFCs [82]. Conversely, BMP9 suppressed proliferation of ECFCs from healthy control subjects. In summary, IPAH ECFCs, like IPAH PAECs, are hyperproliferative. In the case of HPAH, BMPR-II is necessary to inhibit ligand-induced ECFC proliferation.

#### 2.1.5. Venous Thromboembolism (VTE)

VTE is a serious condition that includes deep vein thrombosis and pulmonary embolism [83]. ECs, under physiological conditions, express antiplatelet and anticoagulant molecules to prevent blood coagulation. Although it is known that endothelial dysfunction is key to the initiation and evolution of VTE, a complete understanding of EC actions in the pathogenesis of VTE has not been achieved [84]. Bou Khzam et al. compared the effect of MACs and ECFCs from healthy donors on platelet activity and showed that ECFCs exerted significantly greater inhibitory activity in platelet aggregation than MACs [31]. Moreover, ECFCs constitutively expressed eNOS, cyclooxygenase-1 (COX-1), and COX-2, but conversely, MACs did not. Accordingly, while MAC culture supernatants contained minimal NO and prostacyclin (PGI2), two antiaggregating agents synthesized under the control of NOS and COX, respectively, ECFC culture supernatants had similar levels of NO and PGI2 to those of human umbilical vein ECs (HUVECs) [31]. These data suggest that ECFCs, but not MACs, act like ECs in terms of modulating platelet function. Thus, ECFCs as an alternative to ECs can be used to elucidate the involvement of ECs in the pathogenesis of VTE [31].

Hernandez-Lopez et al. analyzed ECFCs from patients with VTE and reported that VTE ECFCs had markedly lower proliferative potential than control ECFCs [84]. Alvarado-Moreno et al. showed that, although the cell size and capability to form tube-like structures were similar between control and VTE ECFCs, there was a significant increase in reactive oxygen species (ROS) production in VTE ECFCs [85]. Electron microscopy revealed that mitochondrial membrane integrity was disrupted in VTE ECFCs, which was associated with the elevation of ROS, the primary cause of endothelial dysfunction. Additionally, cytokine profiling in VTE ECFCs demonstrated substantially increased production of procoagulative cytokines, most noticeably IL-6, TNF-*α*, and IFN-*γ* [85]. All these cytokines are known to modulate various aspects of thrombosis formation [86–90]. IL-6 has been shown to promote not only megakaryocytopoiesis but also the maturation of megakaryocytes, resulting in an increase in the numbers of platelets [86, 87]. Pignatelli et al. reported that TNF-*α* enhanced collagen-induced platelet aggregation, which was abrogated by a TNF-*α* inhibitor [88]. Pircher et al. showed that TNF-*α* exerted prothrombotic effects through TNF-alpha receptor subtype 2 [89]. IFN-*γ* promotes adhesion of platelets to leukocytes, resulting in platelet activation [90]. Additionally, it has been well documented that elevation of IL-6, TNF-*α*, and IFN-*γ* is linked to coagulopathy seen in a variety of disorders, such as heart failure, COVID-19, and aging [91–96]. These findings suggest that inflammation and oxidative stress are critical to VTE onset and development.

### 2.2. Other Diseases

#### 2.2.1. Diabetes Mellitus (DM)

DM is a chronic, multifactorial metabolic disorder caused by insulin deficiency or resistance resulting in hyperglycemia. DM is classified into type 1 DM (T1DM), resulting from autoimmune-induced *β*-cell death; type 2 DM (T2DM), due to insulin resistance or impaired insulin secretion (that accounts for >90% of diagnosed DM cases); gestational DM; and specific types of DM due to other causes, e.g., chemical-induced diabetes [97]. Hyperglycemia can affect both the macro- and microvasculature leading to end-organ damage that is responsible for the morbidity and mortality seen in DM [98–100].

The first clinical evidence of endothelial dysfunction in DM came to light when impaired endothelium-dependent vasodilation was found in diabetic patients. Jorgensen et al. discovered that healthy controls had increased forearm blood flow after ischemia was induced by arterial occlusion. In contrast, an increase in postischemic blood flow was not seen in patients with T1DM [101]. McVeigh et al. reported that brachial artery acetylcholine infusion led to significantly greater blood flow in the forearm of control subjects compared with T2DM patients [102]. Impaired endothelium-dependent vasodilation in DM patients has since been described by many other groups [103–105]. Studies using animal models of diabetes and those using ECs from healthy human subjects have revealed that hyperglycemia or exposure to high glucose levels stimulates the production of reactive oxygen species (ROS), activates the protein kinase C (PKC) pathway, induces glycation of proteins, increases polyol pathway activity, and promotes proinflammatory cytokine secretion, all of which may contribute to endothelial dysfunction and ultimately vascular complications [106, 107].

To date, a limited number of studies have evaluated ECs derived directly from DM patients. McClung et al. hypothesized that hyperglycemia stimulated EC sloughing. They isolated and enumerated ECs from peripheral blood with anti-CD-146 antibody-coated immune-magnetic beads in T2DM patients and healthy controls. The results showed that patients had a significantly higher number of circulating ECs than healthy controls [108]. Using immunohistochemistry, Galkowska et al. demonstrated increased production of proinflammatory factors, e.g., monocyte chemotactic protein-1 and GM-CSF, and decreased anti-inflammatory cytokines, e.g., interleukin-10, in dermal ECs in the margin of diabetic foot ulcers in patients with T2DM [109]. Others have assessed ECs harvested from DM patients through the abrasion of the intimal surface of a superficial forearm vein with an interventional wire. By analyzing these venous ECs with fluorescent microscopy, Shenouda et al. showed that fission 1 protein levels and mitochondrial fragmentation were significantly increased in T2DM ECs compared with ECs of healthy controls [46]. These findings were confirmed with the *in vitro* culture of commercially available human aortic endothelial cells (HAECs) where exposure to high glucose induced fission 1 expression and augmented mitochondrial fragmentation, leading to increased mitochondrial ROS production, inhibition of acetylcholine-stimulated activation of eNOS, and reduced production of bioactive NO [46]. Tabit et al. reported that although eNOS expression was similar between venous ECs isolated from T2DM patients and nondiabetic individuals, insulin-induced eNOS activation was impaired in T2DM ECs, which was ameliorated by a PKC-specific inhibitor [110]. In addition, both nitrotyrosine, a marker of oxidative stress, and NF-*κ*B, a transcriptional activator promoting the expression of proinflammatory cytokines, were increased in T2DM ECs, as shown by quantitative immunofluorescence [110]. Impaired eNOS activation by insulin in venous T2DM ECs was also described by Bretón-Romero et al. [111]. Of interest, these authors detected an increase in Wnt5a and JNK phosphorylation by quantitative immunofluorescence in T2DM ECs. Further examination with culture of normal HAECs showed that Wnt5a protein activated JNK and abrogated the insulin stimulated eNOS activation [111]. More recently, using low-input RNA sequencing, Beckman et al. performed comparative gene expression profiling of venous ECs and identified 51 significantly upregulated and 101 significantly downregulated genes in T2DM ECs compared with control ECs [112]. Bioinformatics analysis revealed that the mitochondrial metabolic and androgen signaling pathways were impaired, while TGF-*β* and Cyclin-D1 signaling pathways were activated in T2DM ECs [112]. However, the detailed mechanisms of how these altered pathways contribute to endothelial dysfunction remain to be clarified.

**Figure 3:**
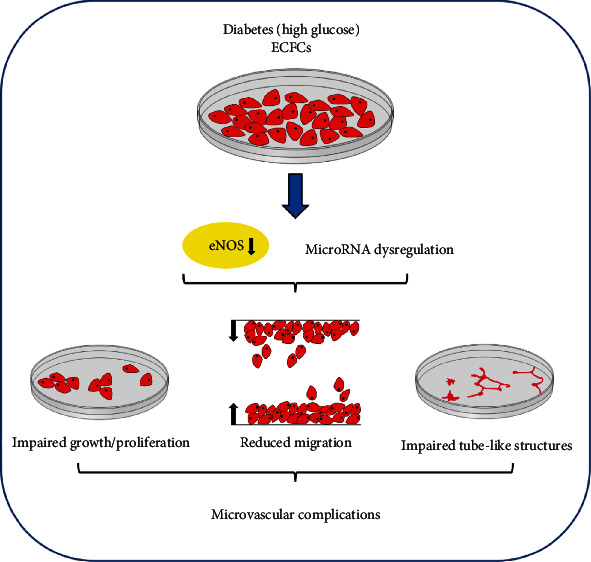
Mechanisms of ECFC dysfunction in the presence of high levels of glucose.

The detrimental effects of high glucose on ECFCs isolated from healthy donors have been described in the literature. Chen et al. found that various aspects of ECFC function that included cell growth, migration, and the formation of tube-like structures were negatively impacted by high glucose and reversed by the addition of a NO donor, but not antioxidants [113]. Western blotting analysis showed that high glucose inhibited eNOS activation that resulted in the reduction of bioavailable NO (Figure 3). These data indicated that NO-related, rather than oxidative stress-mediated mechanisms, are involved in high glucose-induced ECFC dysfunction [113]. Impaired cell proliferation and migration and tube-like structure formation in the presence of high glucose in healthy ECFCs were also described by Wang et al. [114].

The findings of impaired function of healthy ECFCs exposed to high glucose have been corroborated by the functional evaluation of DM ECFCs by several groups. Jarajapu et al. obtained ECFCs in nearly 90% (8/9) of nondiabetic subjects versus in only 30% (3/10) of T2DM patients with microvascular complications (Figure 3), suggesting that hyperglycemia impaired ECFC growth activity [115]. Ho et al. discovered that, compared with healthy ECFCs, T2DM ECFCs formed a substantially lower number of tube-like structures in Matrigel [116]. Langford-Smith et al. also observed decreased proliferation, migration, and tube-like structure formation in T2DM ECFCs, which were associated with reduced NO bioavailability [117]. Leicht et al. investigated the mechanisms underlying decreased T2DM ECFC growth and showed that the level of intracellular superoxide, a major species of ROS, was comparable between T2DM ECFCs and control ECFCs. However, the authors did not measure either eNOS activity or NO production [118]. Wang et al. explored the role that miRNAs play in DM ECFC dysfunction [114] by profiling miRNAs in healthy ECFCs cultured in the presence or absence of high glucose using RNA sequencing. They identified 281 miRNAs significantly upregulated and 167 miRNAs significantly downregulated by high glucose [114]. Two miRNAs, namely, miRs-370 and -134 were also found markedly upregulated in T2DM ECFCs that exhibited impaired growth, migration, and tube-like structure formation compared with control ECFCs (Figure 3). Overexpression of miR-134 in healthy ECFCs had no effect on cell growth but resulted in substantial impairment of cell migration and tube-like structure formation. In contrast, overexpression of miR-370 did not affect ECFC function. Of note, inhibition of miR-134 restored T2DM ECFC function [114]. Bioinformatics analysis revealed that the nuclear receptor-interacting protein 1 (NRIP1), a nuclear protein that modulates transcriptional activity of a variety of transcription factors, is a direct target of miR-134, which was further validated by the luciferase reporter assay. Reduced *NRIP1* expression in T2DM ECFCs was detected by RT-qPCR. Interestingly, healthy ECFCs with *NRIP1* knockdown mimicked T2DM ECFCs in cell growth, migration, and tube-like structure formation [114]. These data suggest that the miR-134-*NRIP1* axis that is dysregulated by hyperglycemia contributes to ECFC dysfunction (Figure 3). This study opens new avenues for research in DM pathophysiology.

#### 2.2.2. von Willebrand Disease (VWD)

VWD is a congenital bleeding disorder caused by mutations in the von Willebrand factor (*VWF*) gene. There are three types of VWD: type 1 VWD is characterized by quantitative VWF deficiency, type 2 is caused by dysfunction of VWF, i.e., qualitative VWF deficiency, and type 3 results from the absence of circulating VWF [119, 120]. VWF, a glycoprotein primarily produced in ECs, present in the multimeric form, is costored with angiogenic and inflammatory molecules in Weibel-Palade bodies (WPBs) [121]. VFW can be secreted constitutively (basal secretion) or upon EC activation. Vascular damage induces the release of VWF that subsequently forms hyperadhesive strings attached to ECs. The VWF strings then bind platelets and the clotting process is initiated [122]. In addition, VWF promotes hemostasis by protecting factor VIII from degradation [122].

A number of studies have investigated VWD pathophysiology using ECFCs. Wang et al. examined type 1 VWD ECFC function and detected significantly lower VWF production and basal secretion in VWD ECFCs compared with ECFCs from healthy donors [123]. Further immunostaining analyses showed the retention of VWF in the endoplasmic reticulum in VWD ECFCs compared with localization of VWF in WPBs in control ECFCs. Although the multimeric pattern of VWF in type 1 VWD ECFCs is largely normal, the agonist-induced VWF secretion and string formation were suppressed in type 1 VWD ECFCs [123]. Such findings were also reported by others [124, 125]. Moreover, Starke et al. found that VWF mRNA downregulation in ECFCs was correlated with lower plasma VWF levels in type 1 VWD [124]. In contrast, type 2 VWD ECFCs have been shown to express normal or elevated VWF mRNA and protein, but with more intracellular VWF retention [124, 125]. For type 3 VWD ECFCs, VWF is not only substantially downregulated, but also negligibly secreted, with minimal or no WPBs in the cells [125, 126]. These data indicate that defective VWF synthesis, storage, and secretion cause VWF deficiency, leading to VWD.

Apart from its hemostatic activity, VWF is also involved in angiogenesis. It was shown that knockdown of VWF in HUVECs by short interfering RNA augmented cell proliferation and migration in the presence of VEGF, and Matrigel plugs implanted in VWF-deficient mice exhibited significantly greater vessel densities compared with those in wildtype littermates [127]. These *in vitro* and *in vivo* findings suggest an antiangiogenic role for VWF. Based on these findings, Starke et al. further studied VWD ECFC angiogenic function and revealed that, compared with ECFCs from healthy donors, type 1 and 2 VWD ECFCs had substantially higher levels of proliferation and migration in response to VEGF and generated more capillary networks as assessed by the *in vitro* Matrigel assay [127]. In contrast, others have reported heterogeneous angiogenic properties of VWD ECFCs [125, 126]. Selvam et al. observed reduced and increased growth rates for type 1 and 2 VWD ECFCs, respectively, in some patients; however, in other patients, growth rates of both type 1 and type 2 VWD ECFCs were similar to those of control ECFCs [125]. Similar migration velocity between type 2 VWD ECFCS and control ECFCs has been described, in contrast to decreased migration velocity for type 1 VWD ECFCs [125, 126]. Type 3 VWD ECFCs demonstrated contradicting migration results, with either reduced or increased migration velocity compared with control ECFCs [125, 126]. *In vitro* Matrigel assays have shown either increased or reduced capability to form capillary networks for types 1, 2, and 3 VWD ECFCs [125, 126].

ANGPT-2, predominantly expressed in ECs, is costored with VWF in WPBs. Selvam et al. showed higher ANGPT-2 mRNA levels in types 1, 2, and 3 VWD ECFCs compared with control ECFCs [125]. Correspondingly, increased ANGPT-2 secretion from across all types of VWD ECFCs were also detected. Using clustered regularly interspaced short palindromic repeats (CRISPR)/CRISPR-associated protein 9 editing technology, Schillemans et al. generated clonal VWF^−/−^ ECFCs and discovered that knockout of VWF resulted in loss of WPBs and elevation in ANGPT-2 release [128]. The necessity for VWF in ANGPT-2 cellular storage was also described by Starke et al. who demonstrated that ANGPT-2 secretion was upregulated in VWF deficient HUVECs [127]. ANGPT-2 is an important angiogenic factor that promotes VEGF-dependent EC sprouting and migration at the early stage of angiogenesis while counteracts ANGPT-1 to inhibit vessel branching and stabilization at the late stage of angiogenesis [69, 70]. Therefore, ANGPT-2 dysregulation leads to abnormal angiogenesis that may be involved in angiodysplasia, the growth of tortuous, dilated, thin-walled mucosal and submucosal venules and capillaries frequently seen in VWD patients [119], which warrants further study.

## 3. ECFCs as Potential Biomarkers for Cardiovascular Disease

Few studies have explored ECFCs as disease biomarkers. Meneveau et al. reported on the growth of ECFC colonies in 40 out of 88 patients with acute myocardial infarction (AMI) at hospital admission [129]. They found that patients with ECFC growth had significantly less microvascular obstruction than those without and that circulating CD34^+^/VEGFR2^+^ cell number was positively correlated with the number of ECFC colonies. Further analysis of the relationship between ECFC production and clinical outcome at 6-month follow-up showed that patients with ECFCs had a smaller territory of infarction, a decreased left ventricular volume and an improved ejection fraction. These data suggest that ECFCs may serve as a potential biomarker for preserved microvascular integrity in AMI patients. Whether the presence of ECFC colonies predicts better long-term outcomes remains to be examined. In view of the scarcity of data in this field, more studies are required to determine the potential for ECFCs as biomarkers for pathophysiological processes.

Extracellular vesicles (EVs) are membrane-bound microparticles that are secreted from virtually all cell types and carry from the parent cell functional proteins and nucleic acids, such as messenger RNAs and miRNAs that mediate intercellular communication [130]. EVs are found in almost every biofluid, and their biomolecular cargo reflects the pathophysiological status of the parent cells, making them attractive biomarkers for the diagnosis and prognosis of diseases, such as cardiovascular diseases and cancers, as summarized in several recent reviews [131–135]. Using flow cytometry to enumerate EC-derived EVs in the peripheral blood of patients with acute ischemic stroke, Simak et al. demonstrated that patients with moderate-severe stroke had a significantly higher number of endoglin^+^, intercellular adhesion molecule-1^+^, or phosphatidylserine^+^ EVs than stroke-free controls. Furthermore, the counts of these EVs correlated significantly with brain lesion volume [136]. These data suggest that circulating EVs are useful biomarkers for the determination of severity and lesion size in stroke. Sinning et al. found significantly higher baseline levels of plasma CD31^+^/Annexin V^+^ EVs in patients with stable CAD who developed major adverse cardiovascular and cerebral events (MACCE), during a median follow-up period of 6.1 years, compared with those without MACCE. Multivariate analysis revealed that baseline levels of CD31^+^/Annexin V^+^ EVs independently predicted MACCE [137]. Human ECFCs produce EVs [138, 139]; however, the potential of ECFC-derived EVs as disease markers has not yet been explored and requires further investigation.

miRNAs have been widely studied for their role in disease diagnosis and prognosis [140–142]. Liu et al. discovered that plasma miR-208a and miR-370 levels were significantly higher in patients with newly diagnosed CAD than in non-CAD controls. The combination of miR-208a and miR-370 detected CAD with a sensitivity of 73.7% and a specificity of 86.0% [143]. Eitel et al. showed that elevated plasma miR-133a levels could serve as a prognostic marker of ST-elevation myocardial infarction; they found that the higher levels of miR-133a conferred greater risk of serious myocardial damage, severe reperfusion injury, and reduced myocardial salvage [144]. Pozo-Agundo et al. demonstrated by RNA sequencing that miR-150 was markedly downregulated in HHT1 and HHT2 plasma exosomes and detected HHT with a sensitivity of 89.4-100.0% and a specificity of 83.9-100.0% [145]. Plasma miR-210 levels have been found to be significantly elevated in HHT patients with pulmonary AVMs (PAVMs) compared with HHT patients without PAVMs or healthy controls. Therefore, circulating miR-210 may be used as a biomarker for the screening of patients with HHT for clinically significant PAVMs [146]. Dysregulated miRNAs in diseased ECFCs have been observed [55, 114], and yet, their diagnostic and prognostic value remains to be appreciated. Of note, extensive studies have shown that circulating CD34^+^/VEGFR2^+^ EPCs are promising biomarkers for cardiovascular diseases, as reviewed by Aragona et al. [147]. Enumerating CD34^+^/VEGFR2^+^ EPCs in the peripheral blood with flow cytometry combined with ECFC assessment may create additive value for disease diagnosis and prognosis, which warrants further study.

## 4. Standardized Methods to Procure, Quantify, and Evaluate ECFCs from Peripheral Blood

Different methods have been employed by different groups to procure, quantify, and evaluate ECFCs, leading to interlaboratory discordances that makes comparison across studies challenging. To address this problem, standardized methods have recently been proposed by the Scientific and Standardization Committee of the International Society on Thrombosis and Hemostasis [148]. The committee recommends Ficoll media with a density of 1.077 g/mL for isolation of PBMNCs from blood collected in either EDTA, citrate, or heparin containing tubes. After isolation, PBMNCs are then suspended in commercially available EGM2 medium containing 10% fetal bovine serum, seeded at a density of 5 × 10^6^ − 5 × 10^7^ per six-well plate coated with fibronectin or collagen, and cultured for 2-5 days until the first change of medium [148]. An ECFC colony is defined as one containing more than 50 adherent cells, and the following results should be reported: (1) the number of ECFC colonies per 10^7^ PBMNCs seeded; (2) the passage number and the population doubling time of ECFCs; (3) the number of days in culture from the day of the PBMNC seeding; (4) number of subjects with zero ECFC colonies; and (5) ECFC function evaluated with appropriate standardized assays [148]. These recommendations will promote ECFC research by increasing data reproducibility and reliability.

## 5. Conclusions

ECFCs, as surrogates for ECs, have been explored to improve the understanding of disease pathophysiology. Generally, lower success of ECFC isolation and abnormal ECFC function are observed in individuals with a pathological condition. Findings from the studies reviewed herein not only support the current knowledge but also provide novel disease mechanisms. Collectively, patient-derived ECFCs appear to be an excellent cell model for the discovery of disease mechanisms, although there is concern that cell reprograming by *in vitro* culture may alter the original “disease state” in ECFCs. Future research should employ larger series of patients and utilize ECFCs isolated with standardized methods at the early and late stages of a pathogenic process to further elucidate mechanisms of disease progression with advanced molecular technologies, such as transcriptomic and proteomic profiling.

## Data Availability

All data are included in the article.
